# Metal-Assisted Injection Spinning of Ultra Strong Fibers from Megamolecular LC Polysaccharides

**DOI:** 10.3390/polym16081099

**Published:** 2024-04-15

**Authors:** Mohammad Asif Ali, Maninder Singh, Shuo Zhang, Daisaku Kaneko, Maiko Kaneko Okajima, Tatsuo Kaneko

**Affiliations:** 1 Key Laboratory of Synthetic and Biological Colloids, Ministry of Education, School of Chemical and Material Engineering, Jiangnan University, 1800 Lihu Ave, Wuxi 214122, China; asifali@jiangnan.edu.cn (M.A.A.); daisaku.kaneko@hotmail.com (D.K.); 2 Graduate School of Advanced Science and Technology, Japan Advanced Institute of Science and Technologies, 1-1 Asahidai, Nomi 923-1292, Ishikawa, Japan; msmanin22@gmail.com (M.S.); tyouseki1215@163.com (S.Z.)

**Keywords:** sacran, fibers, polysaccharide, metal ion, cross-linking

## Abstract

The molecular orientation of liquid crystalline (LC) hydrogels has the potential to induce a range of functionalities that can deliver great mechanical strength. Sacran is a supergiant LC polysaccharide isolated from the cyanobacterium *Aphanothece sacrum* with a high amount of anionic functional groups such as sulfates and carboxylates. In this article, ultra-strong sacran hydrogels and their dried fibers were produced by cross-linking under injection flow with trivalent metal ions such as Al^3+^, Cr^3+^, Fe^3+^, In^3+^, and rare-earth metal ions such Er^3+^ and Sr^3+^. Crossed-polarizing microscopy and X-ray diffraction imaging revealed a uniaxial molecular orientation in the LC gel fiber, resulting in outstanding mechanical characteristics.

## 1. Introduction

Polysaccharides are versatile and abundant renewable biomaterials, but their fabrication is generally difficult in a non-modified natural state [1,2]. Cellulosic yarns are produced from cotton [3]. Regenerated cellulose materials can be manufactured using special and toxic solvents [4,5]. Plastics have also been reportedly fabricated using chemically modified polysaccharides [6,7,8,9]. Starch and pullulan films can be used for water-soluble pharmaceutical capsules and wrappings [10]. Nevertheless, the application of other natural polysaccharides is still restricted.

Metal-binding studies on polysaccharides have been conducted, particularly when concerning the removal of toxic elements from natural water [11] and the preparation of hydrogels [12,13,14,15] for biomedical applications. The binds are based on electrostatic interaction supported by the hydrogen bonding of protonic groups and hydrophilic interactions of polysaccharide chains [5]. Further, electrostatic interaction can be used for various purposes such as anticoagulants or procoagulants, wound dressing, and drug delivery systems. A hydrophobic group can displace interfacial water to improve the wet adhesion of hydrogels through hydrogen bonding [10,11].

Algae-derived polysaccharides (ADPs), such as alginate [1,13], carrageenan, and fucoidan [13] show metal-assisted gelation behavior, which have been extended to food engineering as gels [2,13]. Their metal-binding properties arise from their hydroxyl and other negatively charged functional groups [16,17]. Sodium alginate, an anionic polysaccharide derived from sea algae, is known to have a high gelation ability by binding with divalent metal ions, such as Ca^2+^, in a stationary state [14,15,16].

On the other hand, the spinning of long fibers from hydrogel states has not been attempted, although micro- and nanofibers for unwoven cloths fabricated using appropriate methods (e.g., electrospinning) have been reported [18,19].

The versatile processability of polysaccharides for broad applications requires the development of appropriate conditions for the fabrication of particles, beads, films, or porous hydrogels [10,11]. The functional groups such as carboxyl, hydroxyl, sulfate, phosphate, and amine are responsible for ionic interactions and the further fabrication of polysaccharides [14,15,16]. In addition, we found microalga’s (seaweeds) potential to promote “green” alternatives to petrochemical feedstock [16]. This high water-absorbing green-brown alga called *Aphanothece sacrum* has a huge jelly-like extracellular matrix (ECM) with high water content (97.5–98.3%); their production depends upon the weather and the purity of water [17]. Sacran, which is a cyanobacterial polysaccharide extracted from *A. sacrum* from a jelly-like extracellular matrix growing in local river water, such as underground freshwater in Fukuoka, Japan, is a supergiant anionic polysaccharide with high water retention ability [16,18]. In general, ECMs existing in highly evolved creatures such as mammals are mainly composed of proteoglycan, which are densely aggregated polysaccharides, and fibrous proteins such as collagen, elastin, fibronectin, and laminin [16,18]. Sacran production is now an established industrial process [18]. Sacran chains are composed of 11% of monosaccharides containing sulfate groups and 22% carboxyl groups, and their absolute molecular weight, *M*_w_, evaluated via multi-angle static light scattering (MALLS), is ultrahigh >10^7^ g/mol [18,19]. It is a water-soluble, non-crystalline heteropolysaccharide composed of various sugar residues, such as Glc, Gal, Man, Xyl, Rha, and Fuc, uronic acids, and trace amounts of amino and amphoteric sugars (Figure S1) [20]. *A. sacrum* was classified biologically by Suringar in the late 19th century and has been recognized as a cyanobacterium for more than 100 years [18]. On the molecular scale, sacran chains form rigid rod multi-helix structures showing liquid crystalline (LC) properties with a strong superabsorbent capacity and metal-binding ability [16,19]. The critical concentration of LC sacran is very low (around 0.2 wt%), and they form hydrogels using an extremely high content of water [19,20]. The specific functions of polysaccharides are dominated by their chemical structure with functional groups [18,21] and are important in elucidating the relation among the functional groups of polysaccharides using various ions or chemicals [20,21,22]. In previous works, the salt concentration (NaCl) on the assembly/disassembly of weak microfibers involving both crystalline and amorphous parts was studied under a microscope [20]. The original hierarchical structures of polysaccharides, however, could be difficult to connect with self-assembled structures from the microscopic to macroscopic scale. In contrast, many kinds of environments, which have been used for the understanding of the morphological changes involved in sacran self-assembly through capillary force, drive the geometric deposition of weak microfibrils. Remarkably, unlike typical “microfibrils”, sacrans have diameters ~1 µm and lengths > 200 µm [20,21,22]. However, the most commonly encountered structural motifs in a biofilm are assembly of polysaccharides, with DNA origami representing mega molecules, and cytoskeleton proteins that could self-orient to form LC organizations [22,23]. Hydrophilic networks can be attained via in situ physical and/or chemical cross-linking [21,22]. Physical cross-linking with metallic trivalent cations, such as lanthanoids, promotes gelation behavior in a dilute solution of ~10^−3^ M. In a previous study, we evaluated the extracellular polysaccharides absorbing ions to gelate. The adsorption efficiencies of trivalent lanthanoid ions onto sacran are higher than onto sodium alginate [14,15,16]. In addition, it is quite natural that anionic hydrogels have negative charges in their backbones, which attract positive charges such as amino groups and metal ions. This is due to the ultra-high anionic charge density of sacran (ca. 30,000 charges per chain). [16,17] Moreover, sacran chains form helical rigid-rod assemblies to become liquid crystalline (LC) at a concentration of 0.2 wt%. The critical LC concentration for sacran is much lower than those of other lyotropic biopolymers, such as DNA, cellulose derivatives, xanthan gum, and schizophyllan (>5 wt%) [23]. This clearly shows its high self-orientation ability. Many researchers have mimicked assembled architectures to prepare highly oriented materials, such as hydrogels as soft materials.

However, if amorphous hydrogels are to be used as highly oriented biomaterials, controlling their orientation will be a challenge. Previously, we have reported on the successful casting of sacran films from their LC solutions [20,24]. The study showed that in-plane-oriented films could be obtained. Orientation was seen in the cross-section, but not on the top surface [20]. Moreover, the anisotropic swelling behavior of sacran gel absorbing a lot of water molecules made the sacran chain oriented based on the LC phase. The sacran film has a layered structure with an in-plane orientation of sacran chains. The practical application of hydrogels has been restricted by their low mechanical strength, which is strongly related to their molecular weight. Although researchers are quite curious about sacran fibers and their mechanical properties, the fibers derived from such a high-molecular-weight molecule have never been formed successfully [14,15,17].

In this study, we obtained super strong LC gel fibers of metal ion–sacran complexes by injecting sacran LC solution into various metal ion solutions. After drying the hydrogels, strong fibers with a high orientation degree formed. The cation-assisted gel spinning method should be widely applicable to many other polysaccharides gelating with metal ions.

## 2. Experimental

### 2.1. Materials

*Aphanothece sacrum* was obtained from Green Science Material Inc. (Kumamoto, Japan) and used as received. Ltd. Aluminum (III) chloride (99.9%) was purchased from Tokyo Chemical Industry Co, Ltd., Tokyo, Japan. Scandium (III) trifluoromethanesulfonic acid (99.9%), Chromium (III) chloride hexahydrate (99.9%), and Iron (III) chloride hexahydrate were purchased from Wako Pure Chemical Industry Co, Ltd., Tokyo, Japan. Indium (III) chloride hydrous was purchased from Kojundo Chemical Laboratory Co, Ltd., Sakado, Japan. Yttrium (III) chloride hexahydrate (99.9%), Lanthanum (III) chloride hexahydrate (99.9%), Cerium (III) chloride heptahydrate (99.9%), Neodymium (III) chloride hexahydrate (99.9%), Samarium (III) chloride hexahydrate (99.9%), Gadolinium (III) sulfate octahydrate (99.9%), Dysprosium (III) chloride hexahydrate (99.9%), Holmium (III) chloride hexahydrate (99.9%), and Erbium (III) chloride hexahydrate (99.9%) (Nippon Yttrium Co, Ltd., Omuta, Japan) were used as received without further purification. All other chemicals and solvents were of analytical reagent grade.

#### Extraction of Sacran

Sacran is a macromolecular polysaccharide that was extracted from the extracellular matrix by following a previously described procedure [14,15,18]. Biomaterial samples were washed in pure water to remove soil, grass, and pigments (phycobiliprotein). Further, the washed samples were soaked in isopropanol overnight and then collected via filtration. The isopropanol-washed samples were added to an aqueous solution of 0.1 M NaOH at 100 °C and agitated until completely dissolved. The aqueous solution was further neutralized until a pH of 6–7. Subsequently, the concentrated solution was filtered with cloth to remove undissolved impurities. Further, the viscous solution was slowly poured into pure isopropanol to precipitate white fibrils. The fibrils were collected and washed with isopropanol several times to eliminate impurities. The fibril sacran was further dried at 60 °C for 2–3 min. The average molecular weight of the sacran used in the experiment was measured via size exclusion chromatography with multi-angle light scattering (*M*_w_, 1.6 × 10^7^ g/mol) (*M*_w_/*M*_n_ = 1.07).

### 2.2. Injection Spinning

#### 2.2.1. Preparation of Sacran Solution

Sacran solution (0.5 wt%) was produced by dissolving sacran (0.5 g) in 100 mL of deionized water. The solution was heated at around 70 °C with agitation for more than 24 h. The sacran solution was centrifuged at 20,000 rpm to remove bubbles and impurities.

#### 2.2.2. Preparation of Metal Ions

Aluminum (III) chloride, Chromium (III) chloride hexahydrate, Iron (III) chloride hexahydrate, Indium (III) chloride hydrous, and rare-earth metal were dissolved in distilled water with agitation for 1 h at room temperature to prepare 0.01 M metal solutions.

#### 2.2.3. Fabrication of Uniaxially Oriented Sacran Complex Fiber

The prepared sacran solution was injected into a metal solution prepared after injecting a sacran solution into the metal ion solution using a syringe pump with a speed of 25 mL/min. Some narrow lines were confirmed in the metal solution, which is sacran cross-linking with cations. These hydrogel fibers were clamped using tweezers and passed through an acetone solvent, and then they were dried using the drying method of hanging the hydrogel fibers with a glass bar beam and keeping them perpendicular. Finally, fibers were dried in a dehumidifier (relative humidity 37%) at 40 °C for 2 days. Further, these dried fibers swelled in water to form fiber gels.

### 2.3. Measurement

#### 2.3.1. Mechanical Properties

The mechanical properties of the sacran complex fibers at room temperature were measured using a 5 N Instron 3365 machine with a crosshead speed of 0.2 mm/min. Young’s modulus (*E*) of each sample was estimated at the initial inclination of the stress–strain curves. Young’s modulus (*E*) of each sample was calculated using the following equation with the measurements, where *E* is Young’s modulus (Equation (1)), *σ* is the uniaxial stress or uniaxial force per unit surface, and *ε* is the strain or proportional deformation (change in length divided by the original length).

(1) $$E=\frac{\sigma }{\varepsilon }$$

The test process involved placing the sacran complex fibers in the testing machine and slowly extending the fibers until they fracture. The strain, *ε*, was measured using Equation (2):

(2) $$\varepsilon =\frac{∆L}{L_{0}}=\frac{L-L_{0}}{L_{0}}$$

where ∆*L* is the change in gauge length, *L*_o_ is the initial gauge length, and *L* is the final length of the fiber. Further, the engineering stress, σ, was measured using Equation (3):

(3) $$\sigma =\frac{F}{A}$$

where *F* is the tensile force, and *A* is the nominal cross-sectional area of the sacran complex fiber. The machine calculates the measurements as the force increases so that the data points can be graphed into a stress–strain curve.

#### 2.3.2. Degree of Swelling

The degree of swelling of the sacran complex fibers was measured using the following method. The weights of the dry sacran complex fibers were measured before the sacran complex hydrogel fiber formation. The sacran complex hydrogel fibers, swollen and in an equilibrated state, were weighed after the water on the sample surfaces was removed by wiping. The degree of swelling, *q*, was evaluated, where *W*_s_ and *W*_d_ are the weights in the swollen and dry states, respectively, using Equation (4). The values of 5 specimens were averaged.

(4) $$q=\frac{W_{s}}{W_{d}}$$

#### 2.3.3. Observation of Orientation of Sacran Complex Fibers Using Crossed-Polarizing Microscope (PLM)

Crossed-polarizing microscopy was used to observe the orientation of sacran complex fibers using a microscope (BX51, Olympus, Tokyo, Japan) equipped with a CCD camera (DP80, Olympus). A specimen was cut to size for microscopic observation and placed on the glass slide at room temperature. A first-order retardation plate (530 nm) was inserted into the light path to identify the orientation direction.

#### 2.3.4. Observation of Sacran Complex Fibers Morphology Using Scanning Electron Microscope (SEM)

A scanning electron microscope (SEM, JEOL, JCM-6000PLUS, Tokyo, Japan) was used to investigate the sacran complex fiber morphology. The samples were mounted onto metal stubs using carbon tape. The stubs were then coated with Pt-Pd using a sputtering machine. Energy-dispersive X-ray spectroscopy (EDS) is an elemental analytical technique used for the chemical characterization of a sample.

## 3. Results and Discussion

### 3.1. Fiber Spinning

Sacran was extracted using a previously described method [18,19]. Here, the creation of sacran fibers was attempted using three methods (Figure S2). (1) Fiber formation was fabricated via dry spinning at 200 °C atmosphere using a high-sacran-concentration solution (30 wt%) prepared by dissolving freeze-dried sacran foam in water. As a result, a long fiber was not formed. (2) Fiber formation was attempted using the sacran solution condensed through a slow addition of acetone onto the sacran aqueous solution. Although the aqueous layer was highly condensed, sacran fibers were not obtained from the layer because of the too-high elasticity, as shown in Figure S3. Another solvent, such as ethanol, was used, but the result was not successful. The sacran formed a weak film after casting over the aqueous solution. Sacran fibers, however, were not formed without metal ions. (3) The sacran solution was injected into the solution of various trivalent metal ions to form a physically cross-linked sacran gel fiber (Figure S4) and then dried at 40 °C. The third method succeeded in forming strong long sacran fibers, as described later, and detailed procedures are shown below. The sacran was dissolved in distilled water and heated at ca. 60 °C, with agitation and for more than 24 h, until a viscous sacran solution was obtained (0.5 wt%). The sacran solution was centrifuged at 20,000 rpm to remove impurities and bubbles. To optimize the fabrication of uniaxially oriented sacran complex fibers, the concentration of the sacran liquid crystalline solution was 0.5 wt%. The prepared sacran aqueous solution (0.5 wt%) was injected using a syringe pump into selected trivalent ion solutions (Al^3+^, In^3+^, Nd^3+^, Sm^3+^, Dy^3+^, Ce^3+^, Gd^3+^, and Fe^3+^) to form hydrogel fibers floating in the metal ion solution. The hydrogel fibers were taken out and perpendicularly hung on a rack and dehydrated at room temperature. Finally, the dehydrated fibers were thoroughly dried in a dehumidifier (Figure S5). The fiber’s stability can be affected in the presence of solvent and high humidity. However, we kept the fibers for more than one year wrapped with aluminum foil to confirm no change in the fiber conditions.

### 3.2. Structures

**Figure 1:**
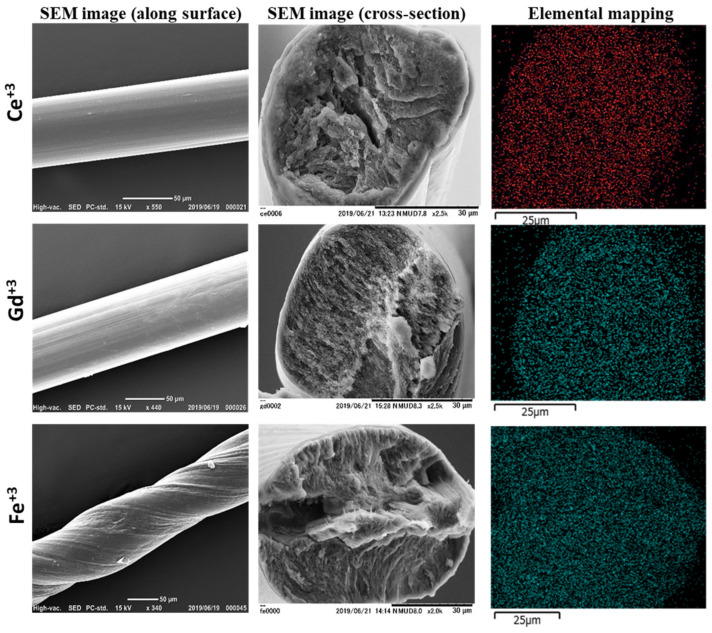
SEM images of surface and cross-sectional parts of the cross-linked sacran fibers with trivalent metal ions (0.01 M). Elemental mapping images along the cross-sections of corresponding trivalent metal ions.

The structural analysis and property evaluation were performed as follows. Figure 1 shows SEM images of sacran complex fibers with Ce^3+^, Gd^3+^, and Fe^3+^ (0.01 M), where fiber surfaces are in the left column and cross-sections of fractured surfaces in the middle column. Other SEM images of the fiber with Al^3+^, In^3+^, Nd^3+^, Sm^3+^, Dy^3+^, Ce^3+^, Gd^3+^, and Fe^3+^ are shown in Figure S5 and Figure 1. Energy-dispersive X-ray spectroscopy (EDS) was also performed on the fractured surfaces of the Ce^3+^-, Gd^3+^-, and Fe^3+^-cross-linked fibers (Figure 1, right column), revealing that the metal elements were homogeneously distributed within the fractured area (Figure S6). The EDS results suggest that sacran fibers formed as a result of metal cations’ interaction with the anionic groups of the oriented chains in a concentration of 0.5%, where sacran chains were in the LC state [16]. In previous papers, we have already reported the selective binding of sacran with trivalent metal ions. Here, we tried to use monovalent and divalent metal ions, but no hydrogel lines were formed. We previously reported that metal cations ionically interact with the anionic groups of the polysaccharide chains, enhancing hydrogen bonds, and forming physically cross-linked gels that affect the surface properties of fibers. An injectable hydrogel passes through various cationic solutions, reflecting the prepared fibers’ surface behavior after drying. From SEM images, one can see a striped texture along the fiber axis on the surface. Although some fibers were twisted, external forces were just applied during drying by chance, where the stripe textures were also observed (Figure S5). The stripe can suggest the fiber orientation [23], supported by the phenomenon of Dy^3+^-cross-linked fibers being spontaneously cracked along the fiber axis. Morphological observations of the fractured surface of sacran complex fibers are shown in the middle column of Figure 1. Sacran orientation was not confirmed, as the uniaxial orientation should be as shown in the general fibers.

**Figure 2:**
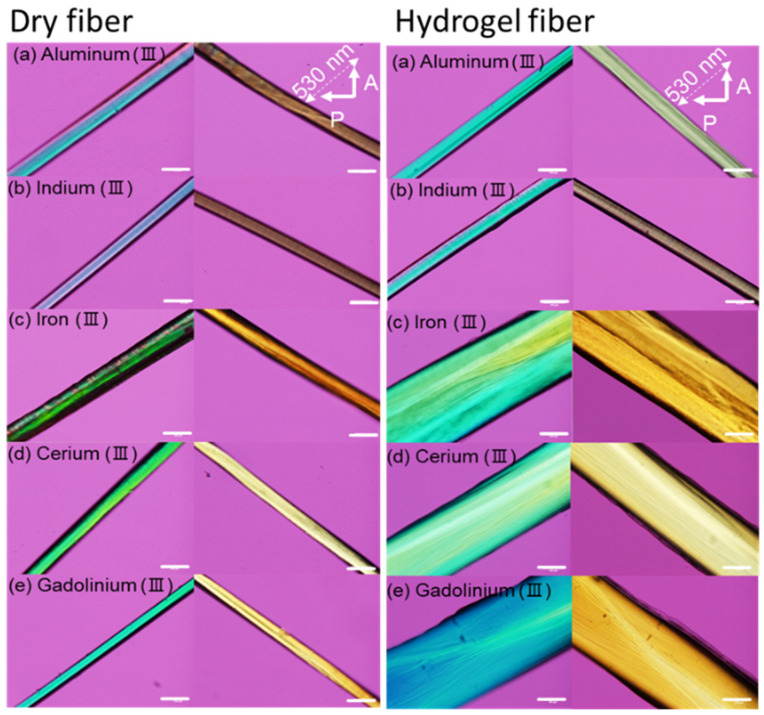
Representative PLM images of sacran complex fibers in dry state and sacran complex hydrogel (swollen state) cross-linked with Al^3+^, In^3+^, Fe^3+^, Ce^3+^, and Gd^3+^. The scale bar is 100 μm.

Fiber orientation was further examined using a crossed-polarizing microscope (PLM) with a first-order retardation plate (530 nm) (Figure 2, left). The polarized microscopic images exhibiting significant intensities show a clear birefringence of the metal ion–sacran complex fiber. The positive birefringence along the fiber axes was found using the additive retardation (blue color) from the upper right to the lower left and the subtractive one (yellow color) from the upper left to the lower right.

The dried sacran fibers cross-linked with different ions were re-immersed in water for 72 h, to form the hydrogel fibers. The obtained hydrogels showed strong uniform birefringence under the PLM, with an almost homogeneously strong color (Figure 2, right). The images revealed that the orientation behavior of the fibers in the hydrogel state was intrinsically the same as in the dried fibers.

### 3.3. Structural Orientation

**Figure 3:**
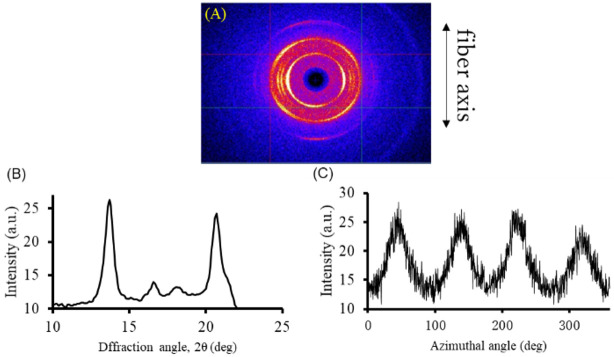
WAXD of sacran complex fibers with Fe^3+^ ions. (**A**) WAXD image. (**B**) Intensity scan as a function of diffraction angle. Two distinct peaks appeared around 2 of 13° and 21°. (**C**) Azimuthal scanning in 2*θ* range of 20.02° and 21.43° was used for calculating orientation degree.

Wide-angle X-ray diffraction (WAXD) studies were performed to investigate crystallinity and molecular orientation. The WAXD images of the complex fibers (Figure 3A) show two arcs on the equatorial line around 13°, 17°, and 18° of 2*θ* diffraction angles, and four arcs around 21°, confirming the molecular orientation. Diffraction should be attributed to the sacran chain networks with metal ions. An intensity profile (Figure 3B) was prepared by scanning the XRD image. Figure 3B shows two distinct peaks around 2θ of 13° and 21°, which imply crystal formation. In a previous study, a sacran dry sheet without any metal binding treatment showed just two broad halo arcs at around 2*θ* = 20°, demonstrating the orientation but no crystallization. The crystal diffraction in the present fibers should be based on the sacran complexation with metal ions. Azimuthal scans (*φ* 0–360°) were performed for the characteristic reflections of sacran complex fibers in the 2*θ* range of 20.02° and 21.43° (Figure 3C). Figure 3C shows four distinct peaks. The degree of orientation can be defined as the average orientation of the normal Hermann orientation function, *f*, which was calculated using Equations (5) and (6) as follows:

(5) $$f=\frac{3<cos2\varphi >-1}{2}$$

(6) $$<{cos}^{2}\varphi >=\frac{\int_{0}^{\frac{\pi }{2}}I(\varphi ){cos}^{2}\varphi sin\varphi d\varphi }{\int_{0}^{\frac{\pi }{2}}I(\varphi )sin\varphi d\varphi }$$

where <cos2*φ*> is the average of the squared cosine of the azimuthal angle, and the values of *f* vary according to the orientation of the molecular chain. The value of *f* was estimated to be 0.42, which is in the range of nematic orientation.

### 3.4. Mechanical Properties

The mechanical properties of sacran complex fibers were investigated using stress–strain tests in an elongation mode. The tensile strength at break, *σ*, elastic modulus, *E*, elongation at break, and strain energy density, *U*, are listed in Table 1. The mechanical properties of the sacran complex fibers were measured from a single specimen prepared after the drying of the single hydrogel fibre sample, and the data obtained were all calculated from an average of five specimens.

**Figure 4:**
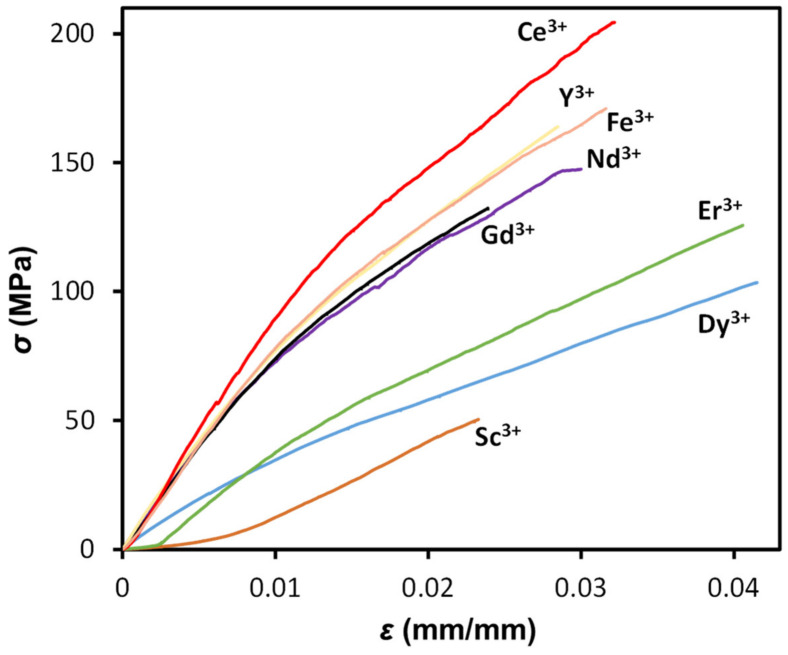
Stress–strain curves of dry fibers of sacran–metal ion complexes with the concentration of 0.1 M.

The *σ* and *E* of the sacran complex dry fibers varied between 0.05 and 0.19 GPa and 2.0 and 5.4 GPa, respectively. Their good mechanical properties can be attributed to their highly oriented structure. The *E* and *σ* of the fiber with lanthanide were higher than those with Sc^3+^. The high values may be due to enhanced interchain interactions with lanthanide contraction. The sacran complex fibers with Ce^3+^ showed an especially high mechanical performance with *σ* = 0.19 ± 0.05 GPa and *E* = 5.4 ± 0.6 GPa (Table 1 and Figure 4) due to the critical gelation concentration of the ions being lower than other lanthanoids metal ions and divalent ions, which means that the sacran affinity with Ce^3+^ was the highest, which might influence the mechanical properties [23]. The value of *E* is higher than those of chemically crossed-linked hydrogels derived from dextrin, chitosan/collagen, natural silk proteins, hyaluronic acid, and cellulose/alginate [21]. The value, *ε*, was in the range of 0.02–0.04, almost independent of the metal cation. The area under the stress–strain curves was used to calculate the strain energy density, which can be regarded as a measure of toughness in materials science. The Ce^3+^-cross-linked fiber showed a much higher toughness than the Sc^3+^ one, with a maximum strain energy density of 5.55 ± 2.06 and 0.73 ± 0.24 kJ/m^3^ because of the smaller size of Ce^3+^ ions. As shown in Figure S6, the mechanical properties of fibers obtained from mixing the sacran solution with CeCl_3_ solutions of varying concentrations (0.001 M to 0.1 M) have also been studied (Table S1). We observed a dependence of σ, E, and U with the CeCl_3_ solution concentration since mechanical properties increased in the 0.001–0.01 M range, but above 0.01 M, an increase in the concentration reduced any mechanical property values (Table S1). Previously, it was reported that the sacran gelation ability decreased at concentrations above 0.01 M. Then, metal-mediated injection spinning might be less effective in a higher concentration over 0.01 M. Then, the dry fiber properties strongly influence the gelation state.

### 3.5. Hydrogels

The hydrogel fibers were prepared after swelling dried fibers in water overnight.

The effects of different metal ion concentrations on the degree of swelling of sacran complex fiber hydrogels were investigated. The swelling degree, *q* (g∙g^−1^), was calculated. The *q* values of sacran complex hydrogel fibers cross-linked using FeCl_3_, GdCl_3_, and CeCl_3_ (0.01 M) were 3, 7, and 10, respectively. However, the sacran network also influenced the *q* values for the different metal ions, not to neglect its impact on the mechanical properties. As shown in the stress–strain curves (Figure S7), the values of σ and *E* for the hydrogel fibers of sacran complex cross-linked with Fe^3+^, Gd^3+^, and Ce^3+^ are very high, ranging from 10 to 39 MPa and 200 to 500 MPa, respectively. Especially mechanical strengths were comparable with reported strong hydrogels such as double-network gels (<21 MPa) [25], nanoclay composite gels (~4.7 MPa) [26], and tetra-PEG gel (~27 MPa) [27]. The high strength is presumably due to the fibrous orientation of sacran chains having an ultra-high molecular weight.

## 4. Conclusions

We successfully prepared uniaxially oriented sacran complex fibers using the ionic interactions between metal cations with anionic supergiant chains in the LC state. A sacran LC solution was injected into different metal ion solutions at varying concentrations, including Al^3+^, Cr^3+^, Fe^3+^, In^3+^, and rare-earth metal ions, to form fibrous LC gels. The sacran complex fibers were formed by drying the gel. The morphological observations via SEM showed a striped surface, and EDS of the fractured surface revealed that the metal elements were homogeneously distributed within the fractured cross-sections. The orientation of the fibers was confirmed using a PLM, showing strong and uniform birefringence. Their orientation degree was evaluated as *f* = 0.42 through WAXD imaging, which is in the range of a nematic LC (0.4~0.7). These fibers/complexes showed excellent mechanical properties. The resulting sacran complex fibers with the highest *E*, at 5.4 ± 0.6 GPa, were Ce^3+^-cross-linked fibers, and *σ* was 0.19 ± 0.05 GPa and *ε*, 0.03 ± 0.01 mm/mm. Excellent mechanical properties were exhibited by super strong hydrogel fibers (the *σ* of gels was 39 MPa at max). Moreover, Sc^3+^-cross-linked fibers showed lower mechanical properties (*σ*: ~0.05 ± 0.1 GPa, *E*: ~2.0 ± 0.4, ε: ~0.05 ± 0.01 with strain energy density 0.73 ± 0.24 kJ/m^3^). In addition, we envision that the research on sacran fibers/complex will be extended to produce mechanically strong materials by drying super strong hydrogels of polysaccharides. This study broadens the research in this field, opening a new avenue for the design and fabrication of sacran complex fiber systems. The strong fibers will be very useful as environmentally adaptive, mechanically reinforcing fillers for biomaterials composed of polymers with hydroxy groups efficiently interacting with polysaccharides, such as poly(hydroxyethyl methacrylate), which is widely used in the dental field. This approach is also capable of constructing additional natural materials toward environmentally adaptive, mechanically reinforcing fillers for bioplastics.

## Figures and Tables

**Table 1:** Mechanical properties of different metal ion-cross-linked sacran dry fibers with a concentration of 0.1 M.

Metal Ions	*E* (GPa)	*σ* (GPa)	*ε* (mm/mm)	*U* (kJ/m^3^)
Sc^+3^	2.0 ± 0.4	0.05 ± 0.01	0.03 ± 0.01	0.73 ± 0.24
Dy^+3^	2.8 ± 0.4	0.11 ± 0.03	0.04 ± 0.01	2.06 ± 0.58
Er^+3^	2.9 ± 0.4	0.12 ± 0.05	0.04 ± 0.02	2.17 ± 0.86
Nd^+3^	4.7 ± 1.8	0.15 ± 0.06	0.03 ± 0.02	2.76 ± 0.91
Gd^+3^	5.2 ± 0.9	0.14 ± 0.03	0.02 ± 0.01	2.15 ± 0.89
Y^+3^	5.4 ± 0.8	0.15 ± 0.05	0.02 ± 0.01	3.69 ± 0.81
Fe^+3^	5.4 ± 0.4	0.17 ± 0.04	0.03 ± 0.01	3.91 ± 0.41
Ce^+3^	5.4 ± 0.6	0.19 ± 0.05	0.03 ± 0.01	5.55 ± 2.06

## Data Availability

Data are contained within the article and Supplementary Materials.

## Supplementary Materials

The following supporting information can be downloaded at: https://www.mdpi.com/article/10.3390/polym16081099/s1, Figure S1: Main structure of sacran which is a supergiant liquid crystalline sulfated polysaccharide; Figure S2: Preparation trial of sacran fibers by dry-spinning in 200 °C atmosphere from 30 wt% aqueous solution; Figure S3: Preparation trial of sacran fibers by jelly-state spinning from gradually dehydrated jelly solutions by stepwise addition of acetone into aqueous solution; Figure S4: Sacran-metal complex fibers. (a) Sacran hydrogel fibers of 0.5 wt% sacran aqueous solution cross-linked with 0.01 M cerium (III) solution by sacran-metal complexation during injection. (b) Sacran-metal complex fibers formed by drying the hydrogel fibers shown in (a); Figure S5: Representative SEM images of sacran complex fibers cross-linked with (a) Al^3+;^, (b) In^3+^, (c) Nd^3+^, (d) Sm^3+^, and (e) Dy^3+^, showing the striped texture on the surface. (f) Spontaneously-fractured fibers of (e); Figure S6: Stress-strain curve of sacran complex fibers prepared under different concentration condition of cerium chloride; Figure S7: Stress-strain curves of sacran-metal complex hydrogel fibers prepared by metal-mediated injection spinning using Ce^3+^, Gd^3+^, and Fe^3+^; Table S1: Mechanical properties of sacran complex fibers prepared under different.
