# Supplementary material for: Metal-Assisted Injection Spinning of Ultra Strong Fibers from Megamolecular LC Polysaccharides
Source: Polymers (Basel). 2024 Apr 15;16(8):1099. doi: 10.3390/polym16081099 (PMC11054878; doi:10.3390/polym16081099)
Supplement: Supplementary file 1 [file polymers-16-01099-s001.zip › polymers-2893991-supplementary.pdf]

Supporting Information for

Metal-Assisted Injection Spinning of Ultra Strong Fibers from Megamolecular LC Polysaccharides

Mohammad Asif Ali [1,2,†], Maninder Singh [2,†], Shuo Zhang [2], Daisaku Kaneko [1,2], Maiko Kaneko Okajima [1,2,*] and Tatsuo Kaneko [1,2,*]

[1] Key Laboratory of Synthetic and Biological Colloids, Ministry of Education, School of Chemical and Material Engineering, Jiangnan University, 1800 Lihu Ave, Wuxi 214122, China; asifali@jiangnan.edu.cn (M.A.A.); daisaku.kaneko@hotmail.com (D.K.)
[2] Graduate School of Advanced Science and Technology, Japan Advanced Institute of Science and Technologies, 1-1 Asahidai, Nomi 923-1292, Ishikawa, Japan; msmanin22@gmail.com (M.S.); tyouseki1215@163.com (S.Z.)
* Correspondence: maiko@jiangnan.edu.cn (M.K.O.); tkaneko@jiangnan.edu.cn (T.K.)
† These authors contributed equally to this work.

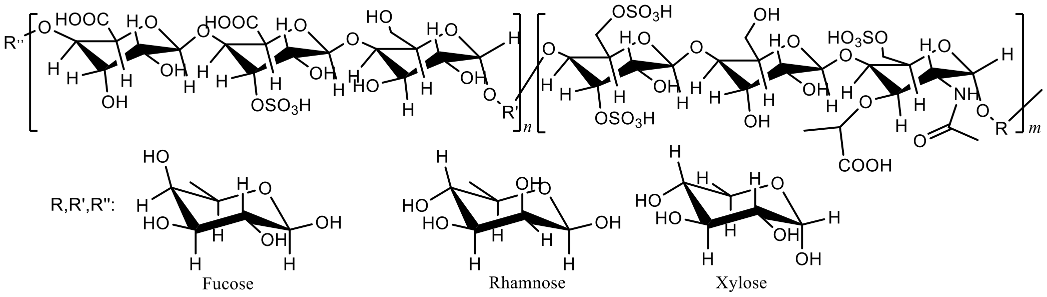

Figure S1. Main structure of sacran which is a supergiant liquid crystalline sulfated polysaccharide.

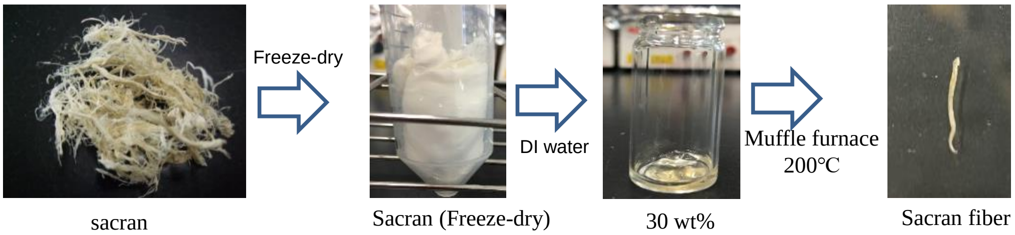

Figure S2. Preparation trial of sacran fibers by dry-spinning in 200 °C atmosphere from 30wt% aqueous solution.

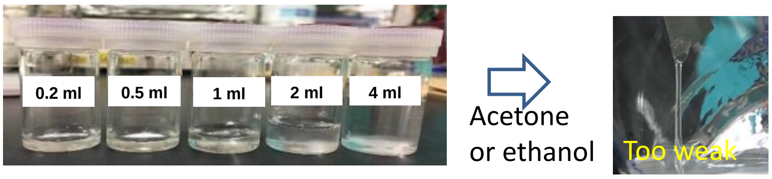

Figure S3. Preparation trial of sacran fibers by jelly-state spinning from gradually dehydrated jelly solutions by stepwise addition of acetone into aqueous solution.

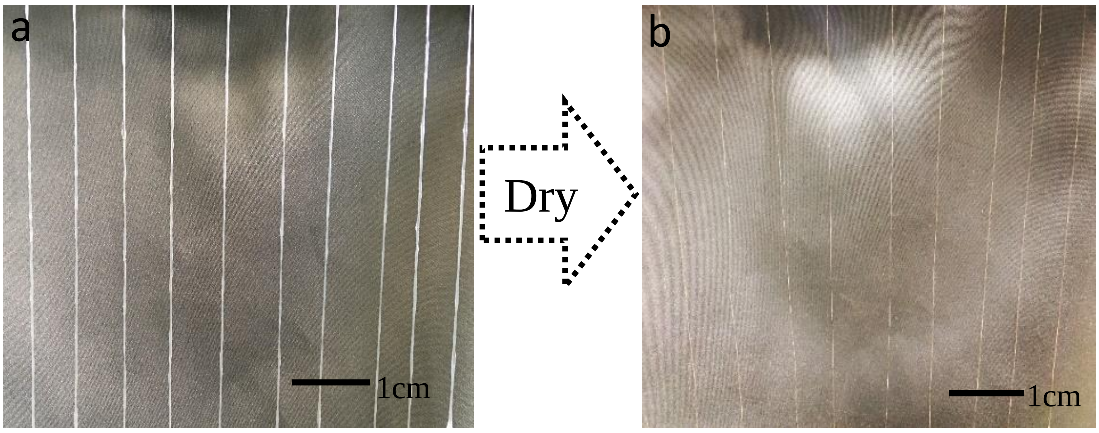

Figure S4. Sacran-metal complex fibers. (a) Sacran hydrogel fibers of 0.5wt% sacran aqueous solution cross-linked with 0.01M cerium (III) solution by sacran-metal complexation during injection. (b) Sacran-metal complex fibers formed by drying the hydrogel fibers shown in (a).

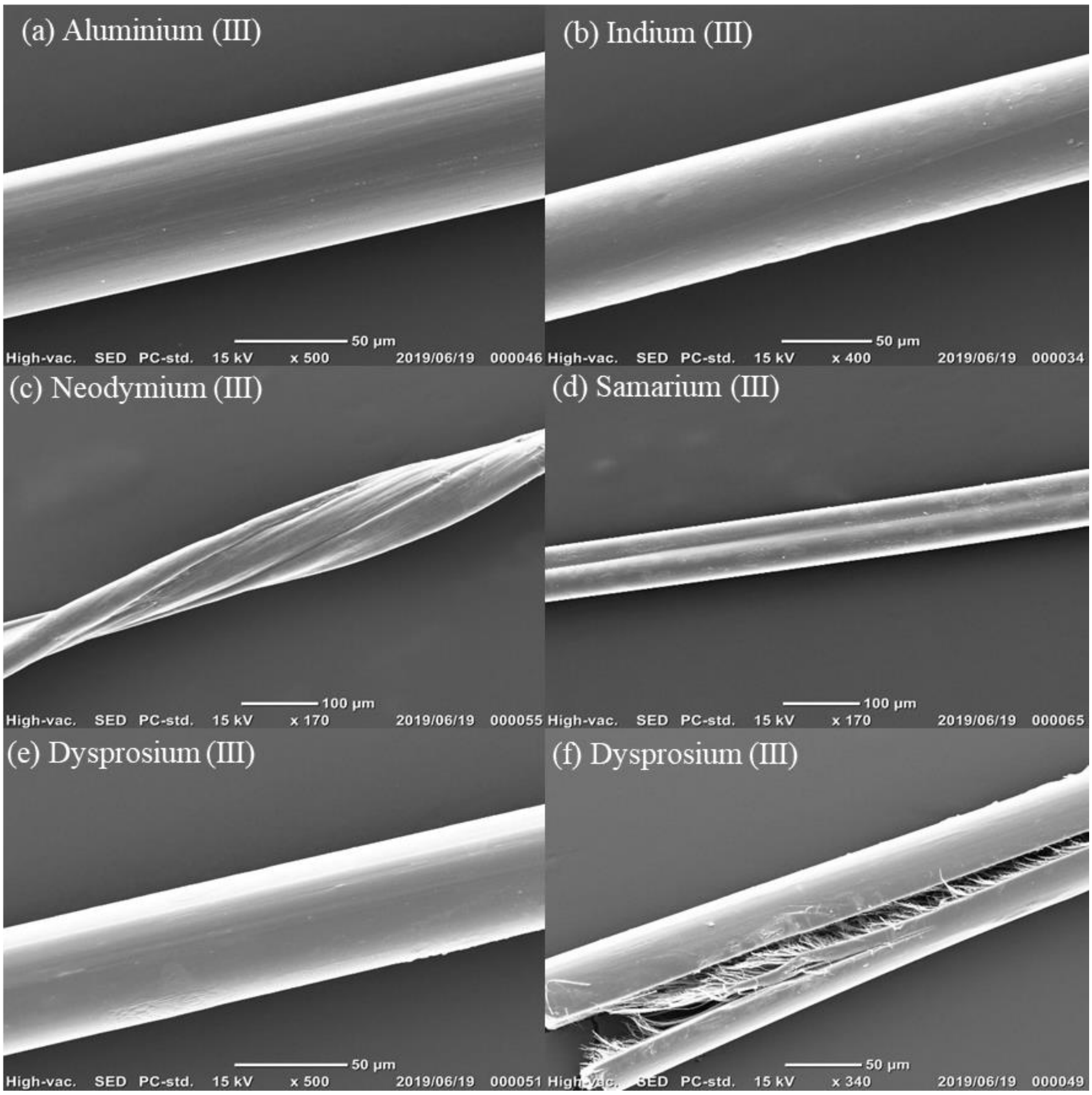

Figure S5. Representative SEM images of sacran complex fibers cross-linked with a) Al^{3+}, b) In^{3+}, c) Nd^{3+}, d) Sm^{3+}, and e) Dy^{3+}, showing the striped texture on the surface. f) Spontaneously-fractured fibers of (e). .

Table S1. Mechanical properties of sacran complex fibers prepared under different concentration condition of cerium chloride.

Concentration of cerium (III) chloride solution	Sacran-cerium complex (III) fibers			
	E (GPa)	*σ* (GPa)	*ε* (mm/mm)	*U* (kJ/m^3)
0.001 M	1.1±0.3	0..09±0.03	0.05±0.02	1.96±1.47
0.005 M	2.3±0.5	0.11±0.03	0.05±0.02	2.92±1.95
0.01 M	5.4±0.6	0.19±0.05	0.03±0.01	5.55±2.06
0.05 M	2.9±0.7	0.09±0.01	0.03±0.01	1.83±0.61
0.1 M	1.9±0.4	0.08±0.01	0.03±0.01	1.65±0.39

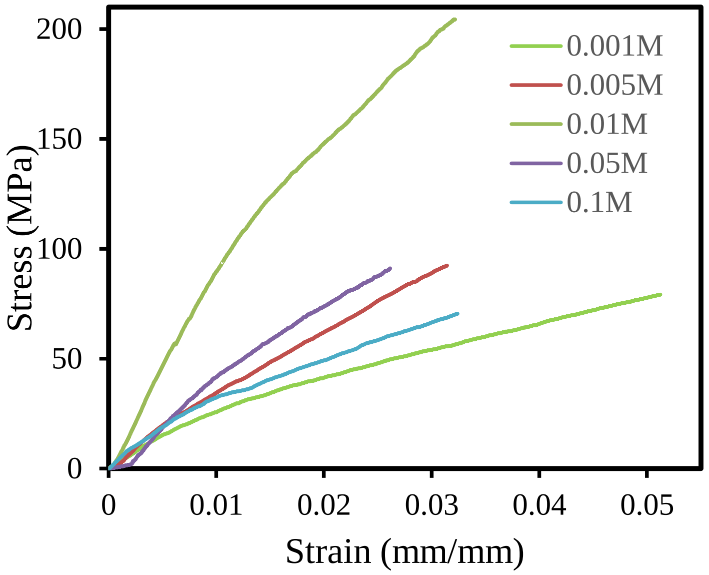

Figure S6. Stress-strain curve of sacran complex fibers prepared under different concentration condition of cerium chloride.

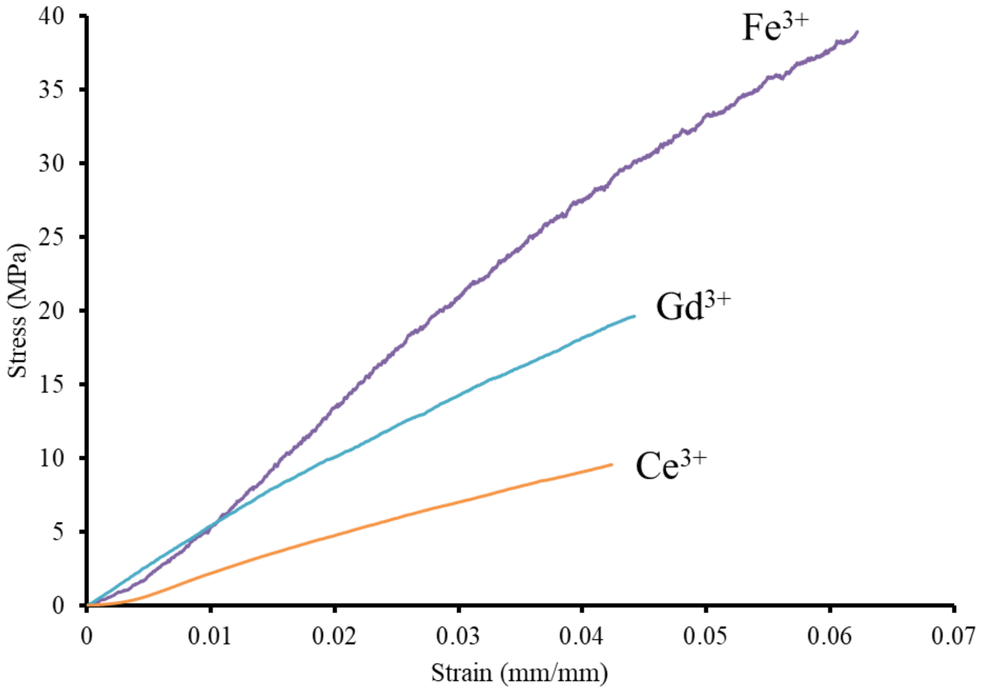

Figure S7. Stress-strain curves of sacran-metal complex hydrogel fibers prepared by metal-mediated injection spinning using Ce^{3+}, Gd^{3+}, and Fe^{3+}.
